# Central Sympathetic Nerve Activation-Mediated Hypertension: Target Mechanisms and Multimodal Interventions—From Basic Research to Clinical Translation

**DOI:** 10.3390/ijms27094063

**Published:** 2026-04-30

**Authors:** Bo Xu, Yi Yang, Renjun Wang

**Affiliations:** Department of Biotechnology, School of Life Science, Jilin Normal University, Siping 136000, China; xubo@mails.jlnu.edu.cn

**Keywords:** hypertension, central sympathetic nervous system, neuroimmunity, epigenetic modification

## Abstract

Hypertension is the leading global risk factor for cardiovascular diseases, and its pathogenesis is closely linked to excessive sympathetic activation, which markedly elevates the risk of stroke, heart failure and other adverse cardiovascular events. Traditional therapies mainly target peripheral mechanisms, whereas the clinical efficacy of renal denervation highlights the critical role of central regulation in sympathetic hyperactivity. This review focuses on the core sympathetic nuclei including the rostral ventrolateral medulla (RVLM) and paraventricular nucleus (PVN), with epigenetic regulation as a key innovative perspective. We systematically summarize the upstream driving effects of reactive oxygen species (ROS) and neuroinflammation, and emphasize lncRNA/miRNA-mediated post-transcriptional regulation and the modulatory actions of gasotransmitters. Under stress conditions, aberrant activation of ROS and neuroimmune pathways, epigenetic reprogramming, and hyperexcitability of central sympathetic neurons act as key events in sympathetic overactivation, which interact synergistically to promote hypertension. Integrating evidence from multiple hypertensive animal models and clinical studies, we discuss multimodal interventions including pharmacotherapy, nanozyme biotechnology and neuromodulation, analyze current translational challenges, and provide a theoretical framework for developing central-targeted antihypertensive therapies.

## 1. Introduction

The pathophysiological progression of hypertension highly relies on excessive activation of the sympathetic nervous system. Functional imbalance of this system constitutes a critical process driving elevated blood pressure and target organ damage, thereby significantly increasing the risk of adverse cardiovascular events and becoming an important factor exacerbating the public health burden of diseases [1,2,3]. For a long time, hypertension therapies have mostly focused on peripheral targets, yet blood pressure control remains unsatisfactory in some patients with refractory hypertension. The clinical efficacy of renal denervation (RDN) has provided a novel perspective for further dissecting the core mechanisms underlying central nervous system-mediated sympathetic hyperactivity, promoting the paradigm shift of hypertension diagnosis and treatment toward central–peripheral coordinated regulation [4,5].

Core sympathetic centers including the rostral ventrolateral medulla (RVLM) and paraventricular nucleus (PVN) serve as integration nodes for stress, inflammatory, and hemodynamic signals, and their dysfunction represents a fundamental basis for central sympathetic activation [6,7,8,9]. Classical studies have established that dysregulation of traditional central and peripheral neurotransmitters and their receptors mediates enhanced sympathetic excitability in hypertension. In recent years, accumulating evidence has revealed that reactive oxygen species (ROS) and neuroimmune inflammation function as upstream drivers, with epigenetic regulation as one of their core regulatory mechanisms, together forming a complex network underlying sympathetic center activation. The innovative core of this review lies in the fact that we have, for the first time, systematically assembled the existing literature evidence linking epigenetic regulation (including DNA methylation, histone modification, and lncRNA/miRNA-mediated post-transcriptional regulation) to gene expression reprogramming, sympathetic neuronal excitability remodeling, and sustained sympathetic hyperactivity in specific sympathetic central brain nuclei (e.g., PVN, nucleus tractus solitarius (NTS), RVLM) under hypertensive conditions. Unlike a general description of epigenetic mechanisms, this review focuses on disease-specific genetic/epigenetic links within these brain nuclei, thereby providing an integrated knowledge framework for understanding the central sympathetic drive mechanisms of hypertension.

This review takes the core sympathetic nuclei as the anatomical framework, with epigenetic regulation and neuroimmune-glial inflammation as the dual innovative themes. It systematically describes the upstream driving role of ROS, dissects the functional links of unfolded protein response, autophagy and mitochondrial homeostasis imbalance in the intracellular regulatory network, and summarizes the central neuromodulatory effects of gasotransmitters. Accumulating evidence indicates that epigenetic reprogramming and abnormal neuroimmune inflammatory loops under stress are key driving events mediating the remodeling of sympathetic central neuronal excitability. These processes interact and amplify synergistically with ROS accumulation and organelle dysfunction, ultimately leading to excessive sympathetic activation and promoting the initiation and progression of hypertension. Based on advances in multiple hypertensive animal models and clinical translational research, this review further summarizes multimodal interventions including pharmacotherapy, nanozyme biotechnology, neuromodulation and nutritional metabolism, and analyzes the critical bottlenecks in translating basic research into clinical applications. This review aims to provide a systematic theoretical basis for novel drug development and clinical strategy optimization of central targeted therapy for hypertension, and facilitate the establishment and improvement of precise diagnosis and treatment systems for refractory hypertension.

## 2. Core Sympathetic Nuclei: Neuroanatomical Substrates for Hypertension Regulation

Although the blood–brain barrier envelops most of the central nervous system (CNS), the choroid plexus, regions of the third and fourth ventricles, and circumventricular organs (CVOs) including the subfornical organ (SFO) and organum vasculosum of the lamina terminalis (OVLT) lack an intact barrier and are classified as periventricular organs (PVOs) [10]. The fenestrated capillary structure of PVOs mediates blood–brain transport of macromolecules and polar molecules, and their ependymal cells interact with cerebrospinal fluid (CSF) and connect nuclei including SFO, OVLT, and area postrema (AP), serving as peripheral signal-sensing units.

RVLM and PVN constitute the core axis of central sympathetic regulation, forming the classic regulatory pathway: PVN–RVLM–spinal cord–peripheral target organs [11,12]. The AV3V region covers periventricular areas, the hypothalamus, and the central part of the preoptic nucleus, extending to the anterior hypothalamus and PVN [13,14]. Studies in Dahl salt-sensitive hypertensive rats have confirmed that AV3V lesion reduces vasopressin secretion and thereby lowers blood pressure [15]. As a core integrative center for physiological functions [7], neurons in the parvocellular region of PVN project directly to RVLM, NTS, and spinal preganglionic sympathetic neurons, participating in the pathological regulation of hypertension and heart failure [9,16,17,18,19,20]. RVLM serves as a key signaling hub for cardiovascular sympathetic regulation, receiving afferent signals from peripheral and central cardiovascular nuclei and activating preganglionic sympathetic neurons in the intermediolateral column (IML) of the spinal cord via descending axons [21,22].

RVLM and PVN form a negative feedback loop with NTS. After integrating baroreceptor signals, NTS modulates the neuronal activity of both nuclei via GABAergic inhibitory projections to maintain blood pressure homeostasis [23,24,25]. Meanwhile, RVLM and PVN receive sympathetic afferent signals from peripheral organs such as the kidney, establishing bidirectional central–peripheral communication. PVN further expands regulatory dimensions through the neuroendocrine axis, constructing a complete neural circuit for blood pressure homeostasis [9,26,27,28].

**Figure 1:**
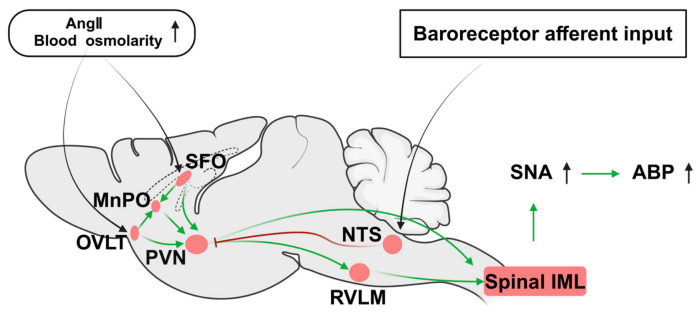
Schematic diagram of the central sympathetic regulatory neural circuit in hypertension. In hypertension, peripheral signals (Angiotensin II, high blood osmolarity) are sensed by circumventricular organs (SFO, OVLT) and relayed to the paraventricular nucleus of the hypothalamus (PVN) via the median preoptic nucleus (MnPO). Baroreceptor afferent input is integrated by the nucleus of the solitary tract (NTS). PVN and the rostral ventrolateral medulla (RVLM) form the core axis of sympathetic regulation, activating preganglionic sympathetic neurons via the intermediolateral column (IML) of the spinal cord to increase sympathetic nerve activity (SNA) and arterial blood pressure (ABP). NTS sends GABAergic inhibitory projections to PVN and RVLM, forming a negative feedback loop for blood pressure homeostasis. In this figure, the red inhibitory bars represent inhibitory projections; the red circles denote key cardiovascular regulatory nuclei; the green arrows indicate excitatory projections; and the black arrows represent sensory inputs. Abbreviations: Ang II (angiotensin II). Created in BioRender. Xu, B.; Wang, R. (2026) https://BioRender.com/jr1nue8.

These brain regions coordinately regulate sympathetic nerve activity in hypertension through specific pathways (Figure 1). Among them, the forebrain pathway is crucial. Due to the lack of an intact blood–brain barrier, SFO and OVLT can efficiently sense peripheral signals such as high salt and angiotensin II (Ang II) [29,30], and relay these signals to PVN via the median preoptic nucleus (MnPO). Magnocellular neurosecretory neurons in PVN promote the release of arginine vasopressin (AVP) from the posterior pituitary gland, increasing arterial blood pressure (ABP) through sodium and water retention; meanwhile, the PVN–RVLM monosynaptic pathway is also involved in sympathetic regulation [31,32]. Neurons in PVN and the PVN–RVLM pathway project signals to preganglionic sympathetic neurons in IML, ultimately increasing peripheral sympathetic activity, inducing vasoconstriction, and elevating ABP [21,22].

In this review, ‘sympathetic nerve activity (SNA)’ refers to the quantifiable electrical activity of sympathetic nerves (e.g., renal SNA), ‘sympathetic outflow’ emphasizes the central-to-peripheral signal transmission, and ‘sympathetic tone’ denotes the basal level of sympathetic drive. These terms are used with these distinctions where appropriate, though they are closely related.

## 3. Molecular Engines of Central Sympathetic Overactivation: From Signaling Pathways to Network Regulation

### 3.1. Central Sympathetic Reprogramming: The Epigenetic Basis for the Onset and Maintenance of Hypertension

#### 3.1.1. DNA Methylation and Histone Modification: Transcriptional Memory of Hypertension-Associated Genes

**Figure 2:**
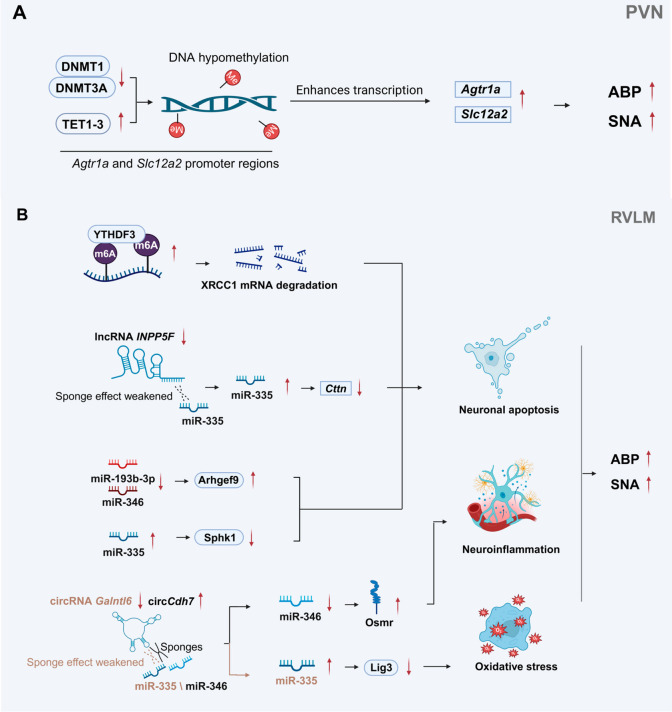
Schematic diagram of epigenetic mechanisms underlying central sympathetic regulation in hypertension. (**A**) Regulatory network of DNA methylation in the paraventricular nucleus (PVN). (**B**) Regulatory network of non-coding RNAs and m^6^A modification in the rostral ventrolateral medulla (RVLM). ↑: Upregulation of expression/activity/function; ↓: Downregulation of expression/activity/function; →: Positive regulation/activation; Sponges: Molecular sponge effect, which attenuates miRNA-mediated suppression of target genes. Abbreviations: DNMT1 (DNA methyltransferase 1); DNMT3A (DNA methyltransferase 3A); TET1-3 (ten-eleven translocation 1-3); Agtr1a (angiotensin II type 1a receptor); Slc12a2 (solute carrier family 12 member 2); BNP (brain natriuretic peptide); SNA (sympathetic nerve activity); circRNA (circular RNA); Galntl6 (polypeptide N-acetylgalactosaminyltransferase-like 6); circCdh7 (circular RNA cadherin 7); miR (microRNA); miR-335 (microRNA-335); miR-346 (microRNA-346); miR-193b-3p (microRNA-193b-3p); Osmr (oncostatin M receptor); Lig3 (DNA ligase 3); Arhgef9 (Rho guanine nucleotide exchange factor 9); Sphk1 (sphingosine kinase 1); lncRNA INPP5F (long non-coding RNA INPP5F); Cttn (cortactin); YTHDF3 (YTH N6-methyladenosine RNA binding protein 3); XRCC1 (X-ray repair cross complementing 1). Created in BioRender. Xu, B.; Wang, R. (2026) https://BioRender.com/71oml1n.

Dynamic remodeling of DNA methylation patterns represents a core mechanism underlying transcriptional reprogramming in sympathetic centers, and its dysregulation plays a pivotal role in hypertension pathogenesis. In spontaneously hypertensive rat (SHR), the promoter regions of *Agtr1a* and *Slc12a2* in the PVN exhibit progressive DNA hypomethylation, driven by a DNMT/TET switch characterized by reduced DNA methyltransferase recruitment and increased enrichment of ten-eleven translocation enzymes (TET1-3). Targeted microinjection of DNMT inhibitors into the PVN mimics the hypertensive phenotype, whereas TET inhibition reverses hypertension and gene overexpression in SHR (Figure 2A) [33]. These findings are consistent with observations in intrauterine growth retardation (IUGR) models, in which PVN DNA hypomethylation activates the sympathetic and renin–angiotensin systems (RAS), confirming that DNA demethylation remodeling in the promoter regions of key central genes is an important epigenetic mechanism mediating central sympathetic overactivation and driving hypertension progression [34].

Histone post-translational modification is an important epigenetic mechanism for the transcriptional regulation of hypertension-associated genes, and its dysregulation can disrupt blood pressure regulatory networks and promote hypertension. Although some studies have not directly focused on central nuclei, the revealed associations between histone modifications, sympathetic activity, inflammation, and sodium metabolism provide valuable insights into central epigenetic regulation of hypertension. Aberrantly elevated histone deacetylases (HDACs) are closely associated with hypertension, and HDAC inhibitors reduce blood pressure in animal models [35]. In salt-sensitive hypertension, high salt inhibits *HDAC8* and enhances histone acetylation via renal β_2_-adrenergic receptors, downregulating *WNK4* and promoting sodium reabsorption [36]. Histone acetylation also regulates norepinephrine transporter (NET) expression and modulates inflammatory factors including TNF-α and IL-6, participating in the comorbidity of depression and hypertension [37,38]. In intermittent hypoxia-induced hypertension, p300/CBP-mediated HIF-1α acetylation activates *Nox2* and *Nox4*, exacerbating oxidative stress and enhancing sympathetic activity [39]. Collectively, these findings demonstrate that histone acetylation modifications contribute to sympathetic overactivation and hypertension by regulating oxidative stress and inflammatory pathways.

#### 3.1.2. RNA Methylation (m6A) Modification: A New Dimension of Epitranscriptomics

RNA methylation (m6A) modification represents an important novel dimension of epitranscriptomic regulation in the sympathetic center (Figure 2B). In the RVLM of spontaneously hypertensive rats, the expression of *YTHDF3*, an m6A reader protein, is significantly elevated. *YTHDF3* promotes the degradation of *XRCC1* mRNA in an m6A-dependent manner, exacerbating neuronal DNA oxidative damage and apoptosis, thereby increasing RVLM neuronal excitability, sympathetic tone, and blood pressure. These findings indicate that aberrant m6A modification serves as a key epitranscriptomic mechanism underlying central sympathetic activation and hypertension [40].

#### 3.1.3. Post-Transcriptional Network of Non-Coding RNAs: Synergistic Regulation of LncRNAs, miRNAs, and circRNAs

In stress-induced hypertensive (SIH) rats, long non-coding RNAs participate in the regulation of sympathetic center excitability via ceRNA networks. In the RVLM of stress-induced hypertensive rats, the expression of *lncRNA INPP5F* is significantly decreased. It acts as a molecular sponge for miR-335 to upregulate Cttn expression and activate the PI3K-AKT pathway to inhibit neuronal apoptosis (Figure 2B). Overexpression of *lncRNA INPP5F* in the RVLM markedly reduces blood pressure, sympathetic nerve activity, and neuronal excitability, suggesting that the *lncRNA INPP5F*/miR-335/Cttn axis is an important non-coding RNA regulatory pathway mediating stress-induced hypertension [41].

MiRNA-mediated post-transcriptional regulatory networks play key roles in the pathogenesis of both neurogenic hypertension and stress-induced hypertension (Figure 2B). In female SHR, miRNA expression profiles in the nucleus of the solitary tract, caudal ventrolateral medulla, and rostral ventrolateral medulla (RVLM) undergo dynamic remodeling from the pre-hypertensive stage to chronic hypertension. Nine core differentially expressed miRNAs form a specific regulatory network by targeting genes associated with angiotensin signaling, neuronal plasticity, and inflammation. This network reprogramming is female-specific, providing important insights into the sexual dimorphism of hypertension [42]. In SIH rats, miR-335 and miR-674-3p are significantly upregulated in the RVLM. Their overexpression elevates heart rate and blood pressure, while knockdown reverses the hypertensive phenotype. MiR-335 targets and inhibits *Sphk1* to promote neuronal apoptosis, forming a pro-hypertensive miR-335/*Sphk1*/apoptosis pathway [43]. In the RVLM of SHR, miR-193b-3p and miR-346 are markedly downregulated. Their overexpression reduces neuronal excitability, sympathetic outflow, and blood pressure. MiR-193b-3p targets and inhibits *Arhgef9* to reduce neuronal apoptosis, exerting protective anti-hypertensive effects that can be reversed by *Arhgef9* overexpression [8].

Circular RNAs (circRNAs) contribute to central sympathetic regulation in hypertension via ceRNA networks (Figure 2B). In the RVLM of SHR, the highly conserved circCdh7 is significantly upregulated. Downregulation of circCdh7 in the RVLM reduces neuronal excitability, sympathetic outflow, and blood pressure. Mechanistically, circCdh7 acts as a molecular sponge for miR-346, and miR-346 targets Osmr, thereby exacerbating astrogliosis and neuroinflammation in the RVLM [44]. In addition, circRNAs are involved in oxidative stress regulation in SIH. In the RVLM of SIH rats, *circRNA Galntl6* is significantly downregulated. Upregulation of *circRNA Galntl6* in the RVLM lowers blood pressure, sympathetic outflow, and neuronal excitability. This circRNA directly sponges miR-335, relieving its inhibition of downstream *Lig3* and thereby attenuating oxidative stress [45]. Collectively, these studies demonstrate that circRNAs mediate abnormal central sympathetic activation via multi-layered ceRNA regulatory axes at the levels of neuroinflammation and oxidative stress, playing critical roles in the pathogenesis of diverse hypertension subtypes.

#### 3.1.4. Model-Dependent Sympathetic Activation Mechanisms

In spontaneous genetic hypertension (e.g., SHR), the central sympathetic hyperactivity is attributed to inherent genetic defects, epigenetic dysregulation, and developmental abnormalities in the PVN and RVLM, which lead to sustained neuronal hyperexcitability. In induced hypertensive models (Ang II, DOCA-salt, stress, renal ischemia), sympathetic overactivation is a secondary adaptive response to circulating hormones, inflammatory factors, oxidative stress, or neural injury. Despite the final common pathway of sympathetic outflow, the upstream triggers, intracellular signaling, and epigenetic regulatory networks differ significantly between genetic and induced models. Therefore, the central mechanisms driving sympathetic nervous system activation are model-specific rather than universal.

Overall, the current understanding of epigenetic regulation in central sympathetic nuclei remains largely limited and preliminary. While DNA methylation in the PVN and RVLM has received relatively more attention, most findings are still based on single or few preclinical studies, with inconsistent results across models. Non-coding RNA networks and m6A modifications are even less explored, and the available evidence remains hypothesis-driven rather than conclusively established. Given the scarcity of consistent central data and the lack of clinical validation, the exact contribution of epigenetic mechanisms to sympathetic overactivation in hypertension still requires further rigorous validation.

### 3.2. Transcriptomic Insights into Central Sympathetic Overactivation in Hypertension

Transcriptomic profiling and high-throughput sequencing have been extensively applied to dissect molecular changes driving central sympathetic overactivation in hypertension. Transcriptome analyses of key cardiovascular regulatory nuclei, including the paraventricular nucleus (PVN) and rostral ventrolateral medulla (RVLM), have identified extensive gene expression remodeling linked to glial activation, neuronal excitability, and inflammatory signaling [8,46,47].

Transcriptomic profiling of microglia in the PVN revealed prominent activation of pro-inflammatory gene networks that enhance central sympathetic outflow in hypertension [47]. Independent miRNA sequencing in the RVLM identified a set of downregulated miRNAs that modulate neuronal excitability and blood pressure [8]. Furthermore, single-nucleus RNA sequencing uncovered distinct astrocyte subtypes that impair glutamate homeostasis and promote neuronal hyperexcitability in hypertension [46]. Collectively, these transcriptomic landscapes highlight critical cell-type-specific molecular alterations that drive central sympathetic overactivation and hypertension.

### 3.3. Neuroimmune Inflammation: A Vicious Cycle of Inflammatory Factors and Glial Cell Activation

#### 3.3.1. Central Proinflammatory Cascade Driven by Inflammatory Mediators

Central inflammation acts as a critical driver of central sympathetic overactivation, whose progression is closely linked to multiple inflammatory mediators and glial activation, collectively participating in the pathological process of hypertension. In the accelerated aging model established by D-galactose (D-gal), aging also induces central inflammation and reduces *klotho* expression in PVN neurons, leading to sympathetic activation and elevated blood pressure, whereas hydrogen sulfide (H_2_S) exerts antihypertensive protective effects by ameliorating cellular senescence and alleviating inflammation [48]. In Ang II-induced hypertensive rats, IL-17A activates glial cells and signaling pathways including TAK1 and NF-κB in the PVN, promoting sympathetic hyperactivity and hypertension, and synergizes with TNF-α and IL-1β to further exacerbate the condition [49]. Its upstream transcription factor *RORγt* aggravates blood–brain barrier damage and microglial activation by regulating IL-17A production, and *RORγt* inhibition alleviates Ang II-induced hypertension [50]. TNF-α itself centrally upregulates the expression of multiple proinflammatory factors in a dose- and time-dependent manner. Neurons in the brain of Dahl salt-sensitive hypertensive rats exhibit stronger inflammatory responses to TNF-α, suggesting a key mechanism underlying the enhanced pressor response in salt-sensitive hypertension [51]. Furthermore, activation of the NF-κB/NLRP3 inflammasome and pyroptosis pathway in the PVN triggers proinflammatory factor release and oxidative stress, thereby enhancing sympathetic drive and increasing blood pressure, constituting a vital pathological component of central inflammation-mediated hypertension [52].

#### 3.3.2. Glial Cell-Mediated Neuroinflammatory Networks

Dysregulation of glial cell-mediated neuroinflammatory networks is one of the core mechanisms underlying central sympathetic overactivation and subsequent hypertension. Microglia participate in the homeostatic regulation of sympathetic centers via both immune and non-immune pathways (Table 1) [53,54]. Under resting conditions, microglia in the PVN continuously release *PDGFB*, which promotes the expression of the potassium channel subunit *Kv4.3* in neurons via the neuronal *PDGFRα* pathway, thereby inhibiting hyperexcitation of presympathetic neurons. Microglial *PDGFB* knockout or *PDGFRα* blockade markedly induces sympathetic hyperactivity and hypertension, indicating an essential non-immune mechanism for maintaining cardiovascular homeostasis [55]. In stress-induced hypertension, microglial dysfunction further exacerbates the condition. On the one hand, reduced localization of σ-1R on mitochondria-associated membranes in RVLM microglia causes abnormal endoplasmic reticulum–mitochondria communication and elevated mitoROS production, thereby inducing microglial M1 polarization and neuroinflammation; σ-1R activation reverses these abnormalities and lowers blood pressure [56]. On the other hand, downregulated IFN-γ and *CCL2* in the RVLM directly impair microglial synaptic phagocytosis, leading to increased synaptic density, elevated neuronal excitability, and further hypertension [57].

In addition, microglia-derived TNF-α impairs RVLM neuronal mitochondrial function by inhibiting the AMPK-Sirt3 pathway, aggravating sympathetic activation and hypertension [58]. Notably, as a key sympathetic regulatory center, the PVN possesses a unique vascular structure characterized by high capillary density and narrow capillary diameter, rendering it susceptible to hemodynamic disturbances induced by elevated blood pressure. ATP released from blood vessels binds to the microglial *P2Y12* receptor and induces microglial inflammatory activation, representing an early trigger for sympathetic hyperactivity [47]. Meanwhile, stress induces lipid metabolism disorder in the RVLM, and *PLIN2* promotes microglial activation and oxidative/nitrosative stress by downregulating phosphatidylethanolamine synthesis; *PLIN2* knockdown alleviates inflammation and hypertension-related injury [59]. Neuronal *ADAM17* mediates microglial chemotaxis via *CX3CL1*, promoting microglial translocation to GABAergic presynaptic terminals in the PVN, reducing inhibitory input to presympathetic neurons, and thus contributing to the pressor response in salt-sensitive hypertension [60]. Concurrently, the expression of *Sik1* in AVP-positive neurons of the PVN is upregulated after a high-salt diet. *Sik1* deficiency exacerbates salt-sensitive hypertension and induces PVN microglial activation, suggesting that *Sik1* maintains blood pressure homeostasis by regulating neuronal and glial function [61].

### 3.4. Organelle Homeostasis Imbalance: Roles of Endoplasmic Reticulum Stress and Autophagy in Neuronal Excitability

Organelle homeostasis imbalance in sympathetic centers including RVLM and PVN is a key event regulating neuronal excitability and participating in the pathological progression of hypertension, among which the synergistic effects of endoplasmic reticulum stress, aberrant autophagy, and mitochondrial dysfunction are particularly prominent (Table 1). In neurogenic hypertension, endoplasmic reticulum stress is aberrantly activated. The expression of endoplasmic reticulum stress markers such as *GRP78* and phosphorylated PERK-eIF2α is elevated in the RVLM of spontaneously hypertensive rats, and these changes precede the onset of the hypertensive phenotype, suggesting that endoplasmic reticulum stress may act as an upstream driver of hypertension. Stabilizing endoplasmic reticulum stress effectively exerts antihypertensive effects [62,63]. Further mechanistic studies reveal that abnormal endoplasmic reticulum–mitochondria coupling mediated by the endoplasmic reticulum transmembrane protein *PDZD8* exacerbates mitochondrial homeostasis disruption, and its downregulation aggravates neuronal excitability disorder, indicating that aberrant endoplasmic reticulum–mitochondria crosstalk serves as a critical molecular link for sympathetic center dysfunction [64].

Aberrant autophagy closely interacts with endoplasmic reticulum stress and neuroinflammation, collectively contributing to central regulation of hypertension. Excessive autophagy is observed in the RVLM of SHRs, and inhibition of such aberrant autophagy effectively reduces blood pressure [63]. In the PVN of Ang II-induced hypertensive mice, activation of the cGAS-STING pathway leads to blocked autophagic flux, while in SIH mice, the HMGB1/RAGE axis mediates aberrant mitophagy. Both conditions trigger neuroinflammation and sympathetic hyperactivity [62,65,66]. Endoplasmic reticulum stress, aberrant autophagy, and neuroinflammation interact to form a regulatory network, which promotes hypertension by disrupting organelle homeostasis and dysregulating neuronal excitability, providing novel insights for central targeted intervention of hypertension [67].

### 3.5. Membrane Excitability Remodeling: Dynamic Changes in Ion Channels and Receptor Function

In SIH rats, voltage-gated sodium channel *NaV1.6* is aberrantly overexpressed in RVLM neurons, representing an important molecular mechanism mediating central hypertension pathogenesis. Elevated expression of the glutamatergic neuronal marker *VGluT1* and reduced *GAD67* expression in the RVLM of SIH rats suggest that *NaV1.6* participates in central regulation of SIH by modulating glutamatergic neuronal function and remodeling neuronal membrane excitability [68]. Further studies confirm that the upregulation of *NaV1.6* in the RVLM coincides temporally with increases in blood pressure and renal sympathetic nerve activity. *NaV1.6* knockdown markedly reduces blood pressure, heart rate, and sympathetic nerve activity, establishing that this channel plays a key driving role in SIH by enhancing neuronal excitability and promoting sympathetic hyperactivity [69].

In addition to ion channels, receptor-mediated central inflammatory pathways are also critical drivers of sympathetic activation. In spontaneously hypertensive rats, Ang II activates TLR4 via *AT1R*, inducing microglial activation, proinflammatory factor release, and blood–brain barrier disruption in the PVN, RVLM, and NTS [70]. Furthermore, crosstalk between receptors and ion channels modulates PVN neuronal excitability. During calcineurin inhibitor-induced hypertension, the interaction between *α2δ-1* and the NMDAR subunit *GluN1* is enhanced in the PVN, upregulating synaptic NMDAR activity and promoting sympathetic pressor effects. Meanwhile, imbalance of the central renin–angiotensin system also participates in central hypertension regulation. Chronic intermittent hypobaric hypoxia lowers NMDAR activity in PVN presympathetic neurons and reduces sympathetic outflow by balancing the hypothalamic RAS axis, thereby decreasing blood pressure [71]. These findings reveal that synergistic abnormalities of receptors and ion channels constitute an important molecular basis for central membrane excitability remodeling in drug-induced hypertension [72,73].

### 3.6. Reactive Oxygen Species Accumulation: A Core Driver of Neuronal Damage and Excitability Remodeling

Oxidative stress is one of the core molecular mechanisms mediating central sympathetic overactivation and promoting the occurrence and development of hypertension. It is mainly characterized by ROS accumulation and abnormal expression of oxidative stress-related proteins, thereby damaging the function of sympathetic central neurons, enhancing neuronal excitability, and closely interacting with mechanisms such as neuroinflammation and organelle homeostasis imbalance (Table 1) [74,75,76,83,84].

In various hypertension models, mitochondrial damage and excessive ROS production exist in key sympathetic nuclei (such as RVLM, PVN, and NTS). For example, mitochondrial damage and ROS accumulation in the RVLM of stress-induced hypertensive rats can be alleviated by resveratrol through activating the AMPK/Sirt3 pathway, thereby reducing neuronal excitability and blood pressure [74]. In SHR, highly expressed *TSPYL2* in the PVN of spontaneously hypertensive rats exacerbates oxidative stress via the JAK2/STAT3 pathway [75]. In the same SHR model, *CB1R* in the PVN promotes inflammation and sympathetic hyperactivity through the Wnt/β-catenin/RAS pathway [77]. In the NTS of offspring with renovascular hypertension, the increased expression of oxidative stress-related proteins and imbalance of AT1R/ATRAP enhance Ang II-induced sympathetic excitation, while prenatal or postnatal administration of H_2_S can correct these abnormalities and alleviate hypertension [76].

Collectively, these findings indicate that targeted intervention of oxidative stress-related molecules or signaling pathways in different nuclei can effectively alleviate ROS accumulation, improve abnormal neuronal excitability, and ultimately lower blood pressure, further confirming the core driving role of oxidative stress in the central regulation of hypertension.

### 3.7. Central Neuromodulatory and Homeostatic Effects of Gasotransmitters

Gasotransmitters are important neuromodulators of central sympathetic and blood pressure homeostasis, among which H_2_S and carbon monoxide (CO) mainly exert protective effects in the central regulation of hypertension by modulating oxidative stress and neuroinflammation (Table 1).

Endogenous H_2_S is crucial for maintaining sympathetic homeostasis. Activation of its synthase *CBS* can alleviate endoplasmic reticulum stress and sympathetic hyperactivity in the PVN of hypertensive rats, while inhibition of *CBS* exacerbates these abnormalities [78]. Similarly, cystathionine gamma-lyase (CSE) is key to H_2_S synthesis; its gene deletion can significantly reduce H_2_S levels in mice and induce hypertension [79], further confirming the basic antihypertensive effect of H_2_S.

Exogenous supplementation of H_2_S also has therapeutic effects. Central administration of the H_2_S donor NaHS can effectively alleviate Ang II-induced hypertension, reduce sympathetic tone, and inhibit PVN microglial activation, and this protective effect is independent of peripheral H_2_S levels [80]. In the high-salt-induced hypertension model, H_2_S in the PVN improves oxidative stress and sympathetic hyperactivity by upregulating *CBS* and balancing the ratio of pro-inflammatory/anti-inflammatory factors; exogenous H_2_S administration can alleviate the pressor response, while inhibition of endogenous H_2_S exacerbates pathological changes [81].

In addition, CO is also involved in central blood pressure regulation. In the PVN of high-salt hypertensive rats, the HO-1/CO pathway is significantly downregulated, while microinjection of the CO donor CORM-2 can upregulate the expression of antioxidant proteins, reduce ROS accumulation and pro-inflammatory factor levels, thereby decreasing sympathetic activity and blood pressure [85]. In summary, as endogenous gasotransmitters, H_2_S and CO play key roles in maintaining sympathetic homeostasis and antagonizing the progression of hypertension through mechanisms such as anti-inflammation, antioxidation, and alleviation of endoplasmic reticulum stress.

Nitric oxide (NO) is another key gasotransmitter critically involved in central cardiovascular regulation. Three distinct nitric oxide synthase (NOS) isoforms mediate NO synthesis, including neuronal NOS (nNOS, NOS I), inducible NOS (iNOS, NOS II), and endothelial NOS (eNOS, NOS III), among which nNOS is primarily responsible for NO production in central cardiovascular-related neurons [86]. Accumulating evidence indicates that the NO system in the NTS and RVLM participates in the neural mechanisms of hypertension by modulating baroreflex sensitivity and sympathetic outflow [87]. Central angiotensin II type 2 (AT2) receptors facilitate baroreflex regulation of renal sympathetic nerve activity (RSNA) through a mechanism dependent on the NO system, whereas angiotensin II type 1 (AT1) receptors exert inhibitory effects on baroreflex function independently of NO [88]. In neurogenic hypertension, disordered NOS signaling and excessive ROS in the brain stem contribute to impaired baroreflex function and enhanced central sympathetic drive. An imbalance between NO and ROS in the NTS and RVLM promotes sympathetic hyperactivity and elevates blood pressure, while restoration of NO bioavailability can improve baroreflex sensitivity and mitigate sympathoexcitation [87].

### 3.8. The Central Regulatory Role of Other Modulatory Mediators

In addition to classic signaling molecules, emerging mediators such as exosomes, neuropeptides, and metabolites also play important roles in central sympathetic activation and the progression of hypertension by regulating oxidative stress and neuroinflammation.

Extracellular vesicles (EVs) mediate the transcellular transmission of central inflammation and oxidative stress (Table 1). In salt-sensitive hypertensive rats, brain-derived EVs can induce neuronal inflammation, activate NADPH oxidase, and trigger mitochondrial ROS accumulation. Injecting such EVs into the brains of normal rats directly induces neuroinflammation and oxidative stress in the PVN and lateral tegmental (LT) regions, confirming the regulatory role of EVs in central hypertension control [82].

Neuropeptides are involved in central sympathetic activation. In SHR, the expression of corticotropin-releasing factor (CRF) and its receptor CRFR1 in the PVN is significantly increased, which further exacerbates sympathetic hyperactivity [89,90]. Similarly, in rats with obesity-related hypertension, the neuropeptide NMU in the PVN can enhance sympathetic activity and increase blood pressure, jointly forming the neuropeptide basis for central regulation of hypertension [91].

Metabolites exert bidirectional effects on sympathetic regulation. There is a significant central-peripheral difference in the role of acetate: as an ethanol metabolite, acetate activates NMDAR in the CeA-RVLM pathway, increases intracellular Ca^2+^ levels, induces oxidative stress, enhances neuronal excitability, and elevates blood pressure [92,93,94]. In contrast, gut microbiota-derived acetate exerts a protective effect: its level is significantly decreased in the serum of SHRs, and supplementation of acetate can inhibit neuroinflammation in the RVLM, repair glial cell morphology and blood–brain barrier function, thereby reducing sympathetic activity and lowering blood pressure [95].

Other regulatory mediators also contribute to hypertension progression. The traumatic stress model can cause transient hypertension, accompanied by robust sympathetic nervous system activation. Beyond the aforementioned mediators, this response involves elevated circulating catecholamines (epinephrine and norepinephrine) from sympathoadrenal activation, hypothalamic–pituitary–adrenal (HPA) axis hyperactivity with increased cortisol release, impaired baroreflex sensitivity, and systemic pro-inflammatory cytokine (TNFα, IL1β) production. At the central level, stress exposure upregulates the expression of vasopressin, AT1R, and FOSL1 in the PVN, and increases mitochondrial ROS, thereby inducing a hypertension-susceptible state [96]. Collectively, these interconnected neuroendocrine, oxidative, and inflammatory pathways synergistically drive sympathetic overactivity during stress-induced transient hypertension, with the highlighted factors representing key contributors among a broader network of regulators. In addition, the Cntnap2^+^ subtype of astrocytes in the RVLM is upregulated in hypertension; downregulating this subtype can reduce neuronal excitability and sympathetic output by upregulating the glutamate transporter Eaat2, thereby achieving blood pressure reduction [46,97].

In summary, multiple emerging mediators including EVs, neuropeptides, and metabolites regulate central oxidative stress and neuroinflammation through specific signaling pathways, jointly participating in the sympathetic activation mechanism of hypertension and providing new targets for central intervention of hypertension.

Collectively, the mechanisms reviewed in this study, including epigenetic regulation, neuroinflammation, oxidative stress, endoplasmic reticulum stress, and ion channel remodeling, are supported by a growing body of preclinical literature. However, most conclusions are based on limited, heterogeneous, and often single-study evidence. The central mechanisms identified in animal models have not been sufficiently validated in humans, and substantial gaps remain in terms of reproducibility, cross-model consistency, and translational potential. This review emphasizes the most plausible pathways while acknowledging the preliminary and incomplete nature of the current evidence base.

## 4. Multimodal Intervention Strategies Targeting the Sympathetic Center

### 4.1. Updates and Iterations of Drug and Acupuncture Interventions: From Central Receptors to Inflammatory Pathways

**Figure 3:**
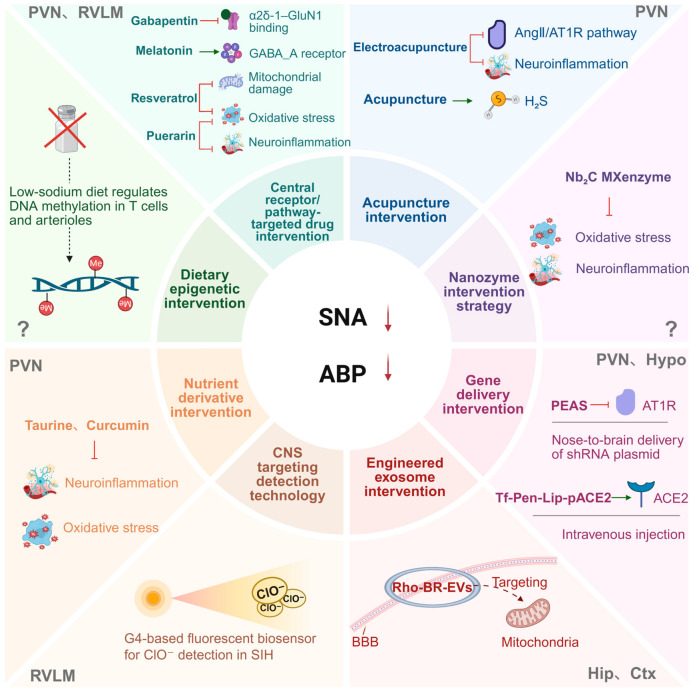
Schematic diagram of multi-modal intervention strategies for hypertension. This figure systematically illustrates multi-modal intervention strategies for hypertension, encompassing pharmacologic & acupuncture interventions, biotechnologic & gene therapies, and nutritional & metabolic interventions. These strategies include both central targeted regulatory mechanisms and select peripheral regulatory pathways, ultimately achieving antihypertensive effects by suppressing sympathetic nerve activity (SNA) and reducing arterial blood pressure (ABP). Whether central nervous system (CNS) involvement is required for certain peripheral interventions remains unclear; thus, question marks (?) are used to denote uncertain central effects. →: Positive regulation/activation/promotion; ⊥: Negative regulation/inhibition/blockade; Downward arrow ↓: Indicates reduction in activity/expression/level; The dashed arrow in the figure represents a regulatory relationship/functional outcome (distinct from direct molecular interactions), indicating that a low-sodium diet exerts a regulatory effect on DNA methylation levels. The cross mark in the figure represents low sodium, and the circle represents α2δ-1. Abbreviations: PVN (paraventricular nucleus); RVLM (rostral ventrolateral medulla); HIP (hippocampus); CTX (cerebral cortex); Hypo (hypothalamus); SNA (sympathetic nerve activity); GABA_a_R (GABA_a_ receptor); α2δ-1-GluN1 (α2δ-1 subunit-GluN1 subunit complex); AngII (angiotensin II); AT1R (angiotensin II type 1 receptor); H_2_S (hydrogen sulfide); Nb_2_C MXenzyme (niobium carbide MXenzyme); PEAS (plasmid DNA encoding angiotensin receptor shRNA); shRNA (short hairpin RNA); ACE2 (angiotensin-converting enzyme 2); Tf-Pen-Lip-pACE2 (transferrin-penetratin modified liposome carrying ACE2 plasmid); Rho-BR-EVs (rhodamine B labeled brain-derived extracellular vesicles); BBB (blood–brain barrier); ClO^−^ (hypochlorite anion); SIH (stress-induced hypertension). Created in BioRender. Xu, B.; Wang, R. (2026) https://BioRender.com/29a3p6c (accessed on 25 March 2026).

Central targeted intervention takes sympathetic core nuclei such as PVN and RVLM as key targets, and inhibits sympathetic excitation and lowers blood pressure by regulating inflammation, oxidative stress, and key signaling pathways (Figure 3).

Regarding the PVN, in salt-induced hypertensive rats, puerarin inhibits the ROS/TLR4/NLRP3 inflammasome pathway [98]; in spontaneously hypertensive rats (SHR), blockade of neuromedin B receptor (NMBR) in the PVN alleviates central inflammation via the RAS/ROS/NF-κB pathway. Additionally, activation of AMPK in the PVN improves renovascular hypertension by inhibiting the ERK1/2-NF-κB signaling pathway [99,100]; in normal Sprague-Dawley (SD) rats, melatonin enhances GABA_A receptor-mediated inhibitory transmission [101], all of which can reduce sympathetic tone. In calcineurin inhibitor-induced hypertension, gabapentin and memantine targeting the α2δ-1-NMDAR pathway, as well as polypeptides interfering with the RCAN1–calcineurin interaction, can effectively alleviate hypertension [72].

Regarding the RVLM, in SIH rats, resveratrol activates the AMPK/Sirt3 pathway to improve mitochondrial damage and oxidative stress [74]; in normal SD rats, blocking the UII/GPR14 pathway can inhibit ERK/N-type Ca^2+^ channel-mediated sympathetic excitation [102]. In SHR, H4R agonists within the RVMM exert central antihypertensive effects by activating GABAergic presympathetic neurons through TRPV1 signaling [73,103].

In SHR, electroacupuncture exerts antihypertensive effects by inhibiting the Ang II/AT1R pathway and inflammatory responses in the PVN [104]; functional microneedle sensors confirm that acupuncture increases H_2_S levels in the PVN, providing direct evidence for its central mechanism [105].

In summary, from drugs to acupuncture, central targeted intervention regulates oxidative stress and neuroinflammation through multiple pathways, providing a new strategy for hypertension treatment.

### 4.2. Biotechnology and Gene Therapy: Precise Delivery and Intervention Strategies

Nanozymes provide a novel approach for hypertension intervention. Two-dimensional Nb_2_C MXene-based nanozymes (Nb_2_C MXenzyme) can efficiently scavenge ROS and inhibit inflammatory factors, and have been verified to exert antihypertensive effects in stress-induced hypertensive rats (Figure 3) [106]. Although the effects of nanozymes are currently observed primarily at the systemic and peripheral levels, they are still included in the discussion given their potent antioxidant and anti-inflammatory activities —activities that may indirectly modulate central sympathetic overexcitation by reducing systemic oxidative stress and neuroinflammatory signals. The direct central effects of nanozymes on sympathetic nuclei, however, remain to be further confirmed.

In terms of gene delivery, nose-to-brain delivery of shRNA plasmids targeting Ang receptors enables efficient hypothalamic enrichment, significantly reducing brain Ang receptor expression and blood pressure [107]. Transferrin and cell-penetrating peptide dual-modified liposomes can cross the blood–brain barrier and target *ACE2* gene delivery to the PVN, alleviating Ang II-induced neurogenic hypertension [108]. Progress has also been made in exosome engineering: brain-derived exosomes can be engineered as mitochondria-targeted delivery systems to precisely transport mitochondrial protectants to damaged neurons and restore mitochondrial function [109]. A preprint study (not yet peer-reviewed) has reported that mitochondria-targeted exosomes constructed via SPAAC click chemistry can accumulate in brain tissue, improve mitochondrial function, and inhibit sympathetic overactivation [110]. Given its preprint status, this finding should be considered preliminary and interpreted with caution, as it has not undergone peer validation. In terms of detection technology, a G-quadruplex DNA-based fluorescent sensor achieves highly sensitive detection of ClO^−^ in the RVLM, confirming ClO^−^ accumulation in the RVLM of stress-induced hypertensive rats, providing a novel monitoring tool for oxidative stress-related diseases (Figure 3) [111].

### 4.3. Nutritional and Metabolic Interventions: Central Regulatory Effects of Dietary Factors

Dietary factors regulate central sympathetic activity and blood pressure through multiple pathways (Figure 3). Taurocholic acid (TCA), a taurine derivative, plays a key role: TCA content is significantly decreased in the PVN of SHR. Bilateral PVN microinfusion of TCA activates the TGR5 receptor, inhibits c-fos^+^ neuronal activation in the PVN, alleviates neuroinflammation and oxidative stress, thereby reducing blood pressure and plasma norepinephrine levels; this effect can be reversed by a TGR5 antagonist [112]. Curcumin exerts central antihypertensive effects via the gut–brain axis: 12-week curcumin intervention in SHRs reduces blood pressure in a dose-dependent manner. The mechanism involves reshaping the gut microbiota, increasing the abundance of butyrate-producing bacteria and plasma butyrate levels, thereby activating the GPR43 receptor in the PVN and attenuating local neuroinflammation and oxidative stress [113].

Dietary sodium intake participates in blood pressure regulation through epigenetic mechanisms: low-salt intervention induces remodeling of DNA methylation profiles in T cells and arteries, with some differentially methylated regions closely related to blood pressure changes. These methylation patterns are conserved between humans and salt-sensitive hypertensive rats, providing a new perspective for the epigenetic mechanism of blood pressure regulation by dietary factors [114].

It should be noted that dietary and metabolic interventions often exert systemic and peripheral effects that may secondarily modulate central sympathetic activity. Although some mechanisms (e.g., sodium-induced epigenetic changes in T cells and peripheral vessels) are primarily peripheral, they are included here because they can shape systemic inflammation, oxidative stress, and hemodynamic signals that ultimately influence central sympathetic nuclei. Thus, these pathways are discussed within a central-targeted framework as upstream modulators of central cardiovascular regulation. In summary, dietary and sodium-related interventions act through both peripheral and central mechanisms. While some epigenetic and metabolic effects occur primarily in peripheral tissues, they indirectly modulate central sympathetic outflow by altering systemic signals. This integrative perspective helps clarify the systemic-to-central cascade underlying blood pressure regulation, justifying their inclusion in a review focused on central sympathetic control.

## 5. Controversies and Bottlenecks: Challenges in Central Targeting Research for Hypertension

### 5.1. Major Gaps in Translational Research: Barriers from Animal Models to Human Application

Studies on the central mechanisms of hypertension rely heavily on animal models (SHR, 2K1C, Dahl salt-sensitive rats, etc.). However, profound differences exist between animals and humans in central nucleus function, neural circuit connectivity, genetic background, and drug metabolism, leading to low efficiency in translating basic research findings to clinical practice. Most intervention strategies achieve precise central administration only in animals, whereas equivalent invasive procedures are difficult to implement in humans. Some non-invasive routes such as intranasal and peripheral administration suffer from insufficient central delivery efficiency and poor target specificity. Furthermore, most animal models are induced by a single etiology, while clinical hypertension is usually multifactorial with overlapping mechanisms, which cannot fully simulate clinical heterogeneity. In addition, the lack of large-scale clinical evidence on long-term safety and individual response variability of central targeted interventions further widens the gap between basic research and clinical practice.

Notably, the mechanisms of central sympathetic overactivation differ between spontaneous genetic hypertensive models and induced hypertensive models. Spontaneous hypertension is driven by genetic–epigenetic programming and inherent neuronal abnormalities, whereas induced hypertension results from environmental or pathological stimuli-driven secondary activation. These differences should be considered in translational research, as therapeutic targets identified in one model may not be equally effective in the other. We would like to note that the current understanding of genetic and epigenetic regulatory mechanisms underlying central sympathetic activation and blood pressure regulation remains incomplete and preliminary. This review only summarizes the currently available evidence from published studies, and further systematic investigations are warranted in the future.

### 5.2. Unresolved Controversies and Research Perspectives: Specificity, Safety, and Long-Term Effects of Interventions

Central sympathetic nuclei are simultaneously involved in multiple physiological functions including cardiovascular regulation, endocrine control, emotion, and stress responses. Targeted interventions are prone to off-target effects, and balancing antihypertensive efficacy with the maintenance of normal physiological functions remains controversial. The expression and function of several central targets differ across hypertension subtypes of varying sex, age, and etiology, and their regulatory mechanisms have not been fully elucidated, limiting the implementation of precision interventions. Currently, most intervention studies focus on short-term antihypertensive effects, lacking systematic evaluation of long-term safety, tolerability, and cardio-cerebro-renal organ protection. Central epigenetic modifications and neuroinflammatory remodeling are persistent and plastic, yet the optimal timing, duration, and combination strategies of interventions remain unclear. Future research should prioritize targeted specificity optimization, long-term safety assessment, and individualized intervention regimens, while clarifying central mechanistic differences across populations, to provide a scientific basis for clinically translatable central targeting strategies.

## 6. Conclusions

The sympathetic central nervous system serves as a dynamic integration center in the pathophysiology of hypertension. Its molecular plastic remodeling is the core mechanism underlying sympathetic overactivation, providing a novel perspective for hypertension research. Mechanisms such as epigenetic networks, neuroimmune inflammation, and abnormal ion channel/receptor function do not exist independently but form a complex regulatory network that collectively mediates central sympathetic activation. Interdisciplinary approaches are crucial for unraveling the complex regulatory mechanisms of the sympathetic center. Future research should break through the bottleneck of translating basic research to clinical practice, develop safe, efficient, and specific multimodal central-targeted intervention strategies, and achieve precise management of hypertension.

## Figures and Tables

**Table 1:** Neuroinflammation and Organelle Homeostasis-Related Molecular Mechanisms in Central Sympathetic Regulation of Hypertension.

Mechanism Category	Key Nuclei	Key Molecules/Pathways	Main Changes in Hypertension	Final Effect	Ref.
Glial cell-mediated neuroinflammatory network	PVN, RVLM	PDGFB/PDGFRα, σ-1R, TNF-α/AMPK-Sirt3, P2Y12, PLIN2, ADAM17/CX3CL1, Sik1	Microglial activation ↑, M1 polarization ↑, synaptic phagocytosis ↓, inflammation ↑	Neuronal excitability ↑, BP ↑	[47,53,54,55,56,57,58,59,60,61]
Organelle homeostasis imbalance	RVLM, PVN	GRP78, PERK-eIF2α, PDZD8, cGAS-STING, HMGB1/RAGE	Endoplasmic reticulum stress ↑, autophagy abnormality, endoplasmic reticulum-mitochondria coupling disorder	Neuronal excitability ↑, BP ↑	[62,63,64,65,66,67]
Membrane excitability remodeling	RVLM, PVN, NTS	NaV1.6, AT1R/TLR4, α2δ-1/GluN1	NaV1.6↑, AT1R/TLR4 ↑, enhanced receptor-channel binding	Neuronal excitability ↑, BP ↑	[68,69,70,71,72,73]
Reactive oxygen species accumulation	RVLM, PVN, NTS	AMPK/Sirt3,mitoROS, TSPYL2/JAK2/STAT3, AT1R/ATRAP, CB1R	ROS↑, mitochondrial damage ↑, oxidative stress ↑	Neuronal excitability ↑, BP ↑	[74,75,76,77]
Gas signaling molecule regulation	PVN	H_2_S (CBS/CSE), CO (HO-1)	Weakened protective effect, inflammation/stress ↑	Neuronal excitability ↑, BP ↑	[78,79,80,81]
Exosomes	PVN, LT	Brain-derived extracellular vesicles (EVs)	Central inflammation↑, oxidative stress ↑	Neuronal excitability ↑, BP ↑	[82]

↑ indicates upregulation of expression/activity/function; ↓ indicates downregulation of expression/activity/function. Abbreviations: Ref. (References); PVN (paraventricular nucleus); RVLM (rostral ventrolateral medulla); NTS (nucleus tractus solitarius); LT (lamina terminalis); PDGFB (platelet-derived growth factor subunit B); PDGFRα (platelet-derived growth factor receptor α); sigma-1R (sigma-1 receptor); TNF-α (tumor necrosis factor-α); AMPK (adenosine 5′-monophosphate-activated protein kinase); Sirt3 (sirtuin 3); P2Y12 (purinergic receptor P2Y12); PLIN2 (perilipin 2); ADAM17 (ADAM metallopeptidase domain 17); CX3CL1 (C-X3-C motif chemokine ligand 1); Sik1 (salt-inducible kinase 1); GRP78 (glucose-regulated protein 78); PERK (protein kinase RNA-like endoplasmic reticulum kinase); eIF2α (eukaryotic translation initiation factor 2 subunit α); PDZD8 (PDZ domain containing 8); cGAS-STING (cyclic GMP-AMP synthase-stimulator of interferon genes); HMGB1 (high-mobility group box 1); RAGE (receptor for advanced glycation end products); NaV1.6 (voltage-gated sodium channel 1.6); AT1R (angiotensin II type 1 receptor); TLR4 (toll-like receptor 4); α2δ-1 (voltage-dependent calcium channel subunit α2δ-1); GluN1 (glutamate ionotropic receptor NMDA subunit 1); mtROS (mitochondrial reactive oxygen species); TSPYL2 (TSPY-like 2); JAK2 (Janus kinase 2); STAT3 (signal transducer and activator of transcription 3); ATRAP (angiotensin II type 1 receptor-associated protein); CB1R (cannabinoid receptor 1); H_2_S (hydrogen sulfide); CBS (cystathionine β-synthase); CSE (cystathionine γ-lyase); CO (carbon monoxide); HO-1 (heme oxygenase 1); EVs (extracellular vesicles); BP (blood pressure).

## Data Availability

The original contributions presented in this study are included in the article. Further inquiries can be directed to the corresponding author.
